# Clinical and biochemical profile among Indian patients with primary hyperparathyroidism

**DOI:** 10.6026/973206300211182

**Published:** 2025-05-31

**Authors:** Manoj Kumar Khandelwal, Shikha Khandelwal

**Affiliations:** 1 Department of Endocrinology, Mahatma Gandhi Medical College and Hospital, Jaipur, India; 2 Department of Nephrology, JNU Medical College and Hospital, Jaipur, India

**Keywords:** Primary hyperparathyroidism (PHPT), clinical profile, biochemical profile, serum calcium, parathyroid hormone, asymptomatic hyperparathyroidism

## Abstract

Clinical and biochemical profile among Indian patients with primary hyperparathyroidism. Hence, a study was conducted from 2022 to 2024 at Mahatma Gandhi Medical College and Hospital, Jaipur, involving 100 patients diagnosed with primary hyperparathyroidism (PHPT). The mean age was 53.2±14.5 years, with a female predominance (69%). Of all patients, 49% were asymptomatic and 51% were symptomatic, with slightly higher mean age in asymptomatic individuals. Biochemically, the mean corrected serum calcium was 12.2±1.6 mg/dl and mean serum parathyroid hormone was 408.9±525.8 pg/ml. A high proportion of asymptomatic cases may reflect improved nutritional status and routine serum calcium screening, highlighting the evolving clinical profile of PHPT.

## Background:

Primary hyperparathyroidism (PHPT) is the disease characterized by hypercalcemia due to autonomous production of parathyroid hormone (PTH) by one or more glands. PHPT is caused by adenoma (85%), hyperplasia (14%) or carcinoma (1%). PHPT is present in 1% of the adult population and its incidence increases to 2% after the age of 55 years in Western series [1]. Classical PHPT, the disorder described by Fuller Albright in the 1930s, was characterized by nephrolithiasis and nephrocalcinosis in over two-thirds of patients and the prototypical skeletal abnormality of the hyperparathyroid state, osteitis fibrosa cystica, was seen in nearly one-third of the affected individuals [2]. The term "asymptomatic primary hyperparathyroidism" was introduced to describe patients who lack obvious signs and symptoms referable to either excess calcium or parathyroid hormone (PTH). In patients with asymptomatic primary hyperparathyroidism, the serum calcium concentration is elevated, but usually only to within 1 mg per deciliter above the upper limit of normal (10.2 mg per deciliter) [3]. In addition, the high prevalence of vitamin D deficiency in these countries may fuel the processes associated with overactivity of the parathyroid glands, leading to more cases of symptomatic disease.

PTH and alkaline phosphatase levels and also greater parathyroid adenoma weight and lower bone mineral density in patients with primary hyperparathyroidism. PTH is the peptide hormone that controls the minute-to-minute level of ionized calcium in the blood and ECF. Although catecholamines, magnesium and other stimuli can affect PTH secretion, the major regulator of PTH secretion is the concentration of ionized calcium in blood [4]. Asymptomatic PHPT is a disease that affects mainly women in their middle years. The disease occurs most commonly within the first decade of the menopause. Women outnumber men by approx 3:1 [5]. Hypercalcemia is mild in asymptomatic PHPT, generally within 1 mg/dL of the upper limit of the normal range. Although it is unknown whether this is true for asymptomatic PHPT, it is clear that patients with mild PHPT and low vitamin D levels have more marked elevations in PTH levels than those who are replete [6]. Therefore, it is of interest to report the clinical and biochemical profile among Indian patients with primary hyperparathyroidism.

## Materials and Methods:

This was a prospective study conducted at Mahatma Gandhi Medical College and Hospital, Jaipur consecutive patients with primary hyperparathyroidism from year 2022 to 2024. The Study was Prospective, cross sectional, observational. Following assumptions have been used in the calculation of sample size Prevalence of asymptomatic patients with primary hyperparathyroidism = 50%Confidence Level = 95% Precision = ± 0.10.

## Inclusion criteria:

Patients with PHPT were included. The diagnosis of PHPT was based on the following criteria:

[1] Elevation of serum calcium above the upper limit of normal range of 10.2 mg /dl

[2] Increased circulatory immunoreactive intact parathyroid hor¬mone (iPTH) above the upper limit of normal range of 68 pg/ml or inappropriately normal for hypercalcemia

## Exclusion criteria:

[1] Secondary or tertiary hyperparathyroidism

[2] Drug-Patients on lithium therapy

[3] Familial hypocalciuric hypercalcemia (FHH)

## Methodology:

## History and examination:

Informed consent was taken from all patients. Patient evaluation included detailed history of symptoms of PHPT including, bone pains/joint pains, bone deformity ,bone swelling, proximal muscle weakness, hypercalcemic symptoms (polyuria, polydipsia, fatigue), renal calculi, pancreatitis, depression and hypertension. This was combined with a food frequency questionnaire to elicit information on compliance towards calcium containing diet. A list of calcium containing food items was prepared and respondents were asked to report the frequency of consumption as daily/ 5-6 times a week/ 3-4 times a week/ 1-2 times a week/ fortnightly/ monthly/ never. The intake was calculated using published food composition table, detailing the nutritive value of Indian foods.

## Biochemical tests:

Biochemical tests were include fasting blood sample for total serum calcium, serum albumin, creatinine, inorganic phosphorus, alkaline phosphatase, intact PTH and 25-hydroxyvitamin D3 (25-OHD).

## Results:

This table shows the mean ± SD age of the patients was 53.2±14.5 years (22-83). Mean ± SD body weight was 64±11.4 kg (39-112), with a mean body mass index of 25.3 ±3.7 Kg/m2 (16.7-37.4). Of the 100 patients, 69 (69%) were females and 31 (31%) were males with female to male ratio of 2.22:1. This was a prospective study conducted from year 2022 to 2024 at Mahatma Gandhi Medical College and Hospital, Jaipur. A total of 100 patients were diagnosed as primary hyperparathyroidism. Table 1 shows the baseline characteristics of all patients. The mean ± SD age of the patients was 53.2±14.5 years (22-83). Mean ± SD body weight was 64±11.4 kg (39-112), with a mean body mass index of 25.3 ±3.7 kg/m2 (16.7-37.4). Of the 100 patients, 69 (69%) were females and 31 (31%) were males with female to male ratio of 2.22:1. The comparison of mean value of various parameters is given in Table 4. The asymptomatic group had a significantly lower mean iPTH level (299.5 vs. 514 pg/mL, P<.05) , significantly lower mean corrected serum calcium level (11.7 vs. 12.7 mg/dl, P<.05), significantly lower mean serum alkaline phosphatase level (118.1 vs. 187.7 U/L, P<.05), significantly lower mean 24-h urinary calcium level (237.5 vs. 294.5 mg/24-h, P<.05) and significantly lower mean adenoma weight (1.87 vs. 3.59 g, P<.05) compared to the symptomatic group. There was no significant difference in any other biochemical variable between the 2 groups. Baseline characteristics of patients with primary hyperparathyroidism (PHPT) including age, gender, height, weight and body mass index (BMI). Values are presented as mean ± SD, median and range (Figure 1 - see PDF). Descriptive statistics of asymptomatic patients with primary hyperparathyroidism. Data include anthropometric measurements, biochemical parameters, urinary indices, post-operative values and parathyroid gland weight. Values are presented as mean, standard deviation (SD), median and range (Table 2). Descriptive statistics of symptomatic patients with primary hyperparathyroidism. Data include anthropometric parameters, serum and urinary biochemical markers, post-operative findings and parathyroid gland weight. Values are reported as mean, standard deviation (SD), median and range (Table 3).

## Discussion:

PHPT is present in 1% of the adult population and its incidence increases to 2% after the age of 55 years in Western series. With the advent of multichannel biochemical screening in the 1970s, the incidence of PHPT increased around the world. Subsequently, the clinical entity of asymptomatic hyperparathyroidism was recognized [9, 10]. There are striking discrepancies around the world with respect to incidence, symptoms and complications of PHPT. In developing countries, particularly India, PHPT is still an uncommonly diagnosed, overtly symptomatic disease of "bones, stones, abdominal groans and psychic moans" [11, 12]. Our study identified a significant proportion of asymptomatic patients (49 %). This high proportion of asymptomatic patients is in contrast to existing Indian studies on PHPT that showed that symptomatic PHPT is the predominant form and accounts for more than 90% of cases . According to a review of Indian PHPT patients, asymptomatic disease is seen only in 5.6% of cases. Our finding is similar to Mishra *et al.* [7] and Zhao *et al.* [13] studies that showed asymptomatic disease in 38% and 38.6% respectively. Bone disease in our study was bone pains in 21 %, polyarthralgia in 14% and fractures in 1%. This proportion of bone disease is in contrast to existing Indian studies on PHPT that showed skeletal manifestations as predominant form and accounts for 77% of cases. Our finding is similar to Mishra *et al.* [7] study where bone disease was 30%. Interestingly, none of the patients had brown tumour or bony deformities, which is again in contrast to the findings of the previous review where bone disease, fractures and brown tumors were present in 77%, 40% and 40% of Indian PHPT patients, respectively. Renal disease was present in 22 % patients. In a review of Indian studies and Mishra *et al.* [7] it was reported in 36% and 20% patients respectively. In developed country it was reported in 15% patients. Pancreatitis was present in 15% patients. In a review of Indian studies it was reported in 15 % patients. In Mishra *et al.* [7] it was 10%. In developed country no patient presented with pancreatitis. The mean corrected serum calcium concentration was 12.2 ± 1.6 mg/dl (10.3-20.3), similar serum calcium profile have been reported in other Indian centers. However, this finding in contrast to Silverberg *et al.*, Rao *et al.* study [5, 6]. This study investigates the relationship between vitamin D levels, seasonal changes, parathyroid adenoma weight, and bone mineral density in patients with primary hyperparathyroidism. It highlights that vitamin D insufficiency is common in these patients and is associated with more severe disease manifestations, although it does not significantly influence adenoma size [8]. In this study mean serum calcium concentration was lower than present study. In present study serum phosphorus concentration was 2.6 ±0.5 mg/dl (1.3-3.9). Similar serum phosphorus profiles have been reported in other Indian centers and west (141-148). In present study serum intact parathyroid hormone concentration was 408.9 ±525.8 pg/ml (79.9-4181).

## Conclusion:

Data shows that 49% patients were asymptomatic. An increasing incidence of asymptomatic PHPT in India is seen. Reasons for high proportion of asymptomatic PHPT is due to better nutritional status of patients from affluent society.

## Figures and Tables

**Table 1:** Baseline characteristics of study patients

	**N**	**Mean**	**SD**	**Median**	**Range**
Age (years)	100	53.2	14.5	53.5	22-83
Gender(F/M)	69/31				
Height (cm)	100	158.9	8	157	143-183
Weight (kg)	100	64	11.4	63.5	39-112
BMI (kg/m^2^)	100	25.3	3.7	25.1	16.7-37.4

**Table 2:** Descriptive statistics of asymptomatic patients

	**N**	**Mean**	**SD**	**Median**	**Range**
Age (years)	49	55.3	12.5	55	26-77
Height (cm)	49	158.4	8.1	156	144-183
Weight (Kg)	49	63.4	12.3	62	39-112
BMI (Kg/M^2^)	49	25.2	4.1	25.1	16.7-37.4
Dietary calcium Intake (mg/day)	49	619.9	188	595	240-1150
Serum calcium (mg/dl)	49	11.7	0.8	11.5	10.3-13.9
Serum phosphorus (mg/dl)	49	2.7	0.4	2.8	1.8-3.8
Serum albumin (mg/dl)	49	4	0.4	4	2.5-4.6
Corrected serum calcium (mg/dl)	49	11.7	0.8	11.6	10.3-13.7
Serum alkaline phosphatase (mg/dl)	49	118.1	52.1	110	49-298
Serum 25(OH)D (ng/ml)	49	19.3	11.4	17.2	2.7-60
Serum iPTH (pg/ml)	49	299.5	329	200.2	79.9-1888
24-h urinary calcium (mg/24 h)	35	237.5	81.3	215	112-470
24-h urinary creatinine (mg/24 h)	35	891.8	238	869	535-1795
Calcium creatinine clearance ratio	35	0.018	0.01	0.017	0.01-0.04
Spot urinary calcium (mg/dl)	14	25.9	6.5	26	13-35
Spot urinary creatinine (mg/dl)	15	76.6	20.6	73	48-122
Spot urinary calcium creatinine ratio	14	0.02	0.01	0.02	0.009-0.034
Post-operative serum calcium (mg/dl)	43	9.1	0.55	9	8.2-10.1
Post-operative serum iPTH (pg/ml)	43	23.6	18.3	17.9	2.6-66.6
Gland weight (g)	43	1.87	1.89	0.8	0.1-6.2

**Table 3:** Descriptive statistics of symptomatic patients

	**N**	**Mean**	**SD**	**Median**	**Minimum**
Age (years)	51	51.1	16.1	51	22-83
Height (cm)	51	159.3	7.9	158	143-173
Weight (Kg)	51	64.5	10.6	65	46-88
BMI (Kg/M^2^)	51	25.3	3.3	25.1	19.1-35.3
Dietary Calcium Intake (mg/day)	51	621.9	200.4	610	280-1175
Serum calcium (mg/dl)	51	12.6	1.9	12.3	10.3-19.6
Serum phosphorus (mg/dl)	51	2.6	0.6	2.6	1.3-3.9
Serum albumin (mg/dl)	51	3.8	0.5	4	2.5-4.8
Corrected serum calcium (mg/dl)	51	12.7	1.9	12.3	10.6-20.3
Serum alkaline Phosphatase (mg/dl)	51	187.7	222.7	122	47-1428
Serum 25(OH) D (ng/ml)	51	22.2	16.7	18	6-86.9
Serum iPTH (pg/ml)	51	514	648.7	266	118.7-4181
24-h urinary calcium (mg/24 h)	42	294.5	112.5	278.5	112-739
24-h urinary creatinine (mg/24 h)	42	944.8	238.2	937.5	460-1751
Calcium creatinine clearance ratio	42	0.022	0.01	0.021	0.01-0.07
Spot urinary calcium (mg/dl)	9	20	3.8	18	16-27
Spot urinary creatinine (mg/dl)	9	50.9	16.5	45	32-80
Spot urinary calcium creainine ratio	9	0.03	0.008	0.03	0.02-0.045
Post-operative serum calcium (mg/dl)	45	9	0.62	9	6.9-10.1
Post-operative serum iPTH (pg/ml)	45	18.2	17.15	12.4	2.0-69
Gland weight (g)	45	3.59	4.45	1.9	0.2-18.5

**Table 4:** Independent Student t - test/Mann Whitney U Test for comparison of mean value of parameters between symptomatic and asymptomatic groups

	**Mean Difference**	**SE Difference**	**95% Confidence Interval of the Difference**		**t - value**	**p - value**
			**Lower**	**Upper**		
Age (years)	-4.23	2.89	-9.966	1.51	-1.462	0.147
Height (cm)	0.89	1.6	-2.293	4.065	0.553	0.582
Weight (Kg)	1	2.3	-3.558	5.562	0.436	0.664
BMI (Kg/M^2^)	0.1	0.74	-1.371	1.58	0.141	0.888
Dietary calcium intake (mg/day)	1.96	38.92	-75.277	79.207	0.05	0.96
Serum calcium (mg/dl)	0.92	0.29	0.345	1.503	3.169	0.002*
Serum phosphorus (mg/dl)	-0.13	0.1	-0.337	0.078	-1.242	0.217
Serum albumin (mg/dl)	-0.15	0.09	-0.33	0.037	-1.585	0.116
Corrected serum calcium (mg/dl)	1.04	0.29	0.456	1.627	3.53	0.001*
Serum alkaline phosphatase (mg/dl)	69.65	32.65	4.859	134.442	2.133	0.035*
Serum 25(OH)D (ng/ml)	2.86	2.87	-2.83	8.554	0.998	0.321
Serum iPTH (pg/ml)	214.49	103.47	9.146	419.824	2.073	0.041*
24-h urinary calcium (mg/24 h)	56.92	22.79	11.522	102.317	2.498	0.015*
24-h urinary creatinine (mg/24 h)	52.99	54.45	-55.479	161.451	0.973	0.334
Calcium creatinine clearance ratio	-0.004	0.002	-0.008	0.0002	-1.87	0.065
Spot urinary calcium (mg/dl)	-4.2	3.22	-10.886	2.486	-1.303	0.064
Spot urinary creatinine (mg/dl)	-20.58	10.32	-41.986	0.83	-1.993	0.015
Spot urinary calcium creatinine ratio	0.008	0.003	-0.15	-0.0004	-2.199	0.039*
Post-operative serum calcium (mg/dl)	-0.63	0.3	-1.233	-0.03	-2.085	0.040*
Gland weight (g)	-1.72	0.735	-3.181	-0.256	-2.337	0.004*
Post-operative serum iPTH (pg/ml)	-5.93	3.74	-13.367	1.513	-1.583	0.117
* p - Value < 0.05,
statistically significant
